# A Rate-Supported Institution

**Published:** 1900-11-24

**Authors:** 


### A RATE-SUPPORTED INSTITUTION.

The following horrible story was told at the Manchester Assizes. We take the account from the *Times* of the 17th inst.

On Wednesday, George Prescott, 26, workhouse attendant, was charged with the murder of Francis Southgate on October 3rd. Southgate was a pauper imbecile in a night ward under the prisoner's charge. According to the evidence for the prosecution, on the evening of October 3rd Southgate was making a great noise, disturbing other inmates, and Prescott in a struggle with him held him down on the floor by the throat, whilst another attendant held his legs. After this Southgate got up and went to bed. Some hours later, about midnight, Southgate was again making a disturbance, when Prescott again took him by the throat and appeared to be throttling him. Later, at half-past two in the morning, Southgate beginning again to be noisy and troublesome, Prescott sent an inmate, who acted as a night helper, for a towel, saying he "knew how to quiet a troublesome man like that." He put the towel in a loop round the neck of Southgate and twisted the ends of the loop together, and so held him down in the bed. Southgate gasped, "You'll hear of this again, Mr. Prescott," and struggled out of his bed upon the floor and under the bed. Prescott then called out for a poker, which was given to him by the inmate assistant, who then went about his duties, but saw Prescott begin to twist the towel tighter by using the poker, as for a tourniquet. Shortly afterwards Prescott was heard to cry out, "Good God! the man is dead," and he went and reported to the doctor that the inmate Southgate was dead. The doctor misunderstood the message as an ordinary announcement not requiring attendance until the morning. The prisoner continued his duties until seven in the morning, when he was relieved. Before going off duty he asked the inmate helper to say nothing. Medical evidence was given to show that, although Southgate was suffering from general paralysis of the insane, the disease was not far advanced, and there was nothing in his ordinary state to account for death. The certificate of death was first given as from cardiac syncope. An autopsy revealed the cause of death as asphyxia, resulting from strangulation. The cartilage of the larynx had been fractured. For the defence expert surgical evidence was given that the marks on the neck of the deceased might have been caused by fingers, and were not likely to have been caused by a towel. The untrustworthiness of the evidence of workhouse inmates compelled to do duty in night wards and the excellent character of the prisoner were also urged as grounds on which the jury might find the prisoner guilty only of the less grave charge. The Judge carefully directed the jury upon the two issues which were open to them. After half-an-hour's retirement the jury returned a verdict of guilty of manslaughter, and the prisoner was sentenced to seven years' penal servitude. The case created very great local interest.

We do not wonder that this case excited "great local interest.' The fact is that it is of far more than mere local interest, for it must be taken as showing what is possible under the spell of that deadly officialism which seems to take root and thrive with especial readiness in workhouse institutions. Wherever the sick and the helpless are shut up and are handed over to the tender mercies of a purely official class; whenever the doors are locked; whenever outside inspection is kept at arm's length and public criticism is regarded as an impertinence, there abuses are apt to creep in, and can indeed only be kept out by exceptional care on the part of those in high authority; and although in many workhouse hospitals, and even in many workhouses, everything is done in proper order, and the humane element is allowed free play, no one can doubt that the system on which these institutions are managed is such as to give much encouragement to that greatest cause of misery to the poor, the tyranny of minor officials. Those who remember the revelations made in regard to workhouse management by the late Dr. Anstie, Dr. Rogers, and others, the agitation to which they led, and the reforms which in consequence of them were set on foot, and who remember that this was more than thirty years ago, must be shocked to find that such things as are described above can still happen even at the present day. That a violent-tempered attendant should illtreat or even kill a patient is, perhaps, a thing that might happen anywhere. It is bad enough, but after all it is but a failure of the human instrument. What, however, is shocking is that the system should have been such that this prolonged and repeated torture of an unfortunate lunatic, even to his death, should have gone on without causing such protests as to have brought it to a sudden end; that in a public institution it should have been possible for the death of a man who was not expected to die to be reported to the medical officer, and that yet for the announcement to be regarded as a mere formal matter which would do in the morning; that on a patient dying in such circumstances a certificate of death from "cardiac syncope" should have been given; that while the tragedy was being enacted in the ward the ward assistant should be found "going about his duties," apparently as usual; and that even the prisoner, knowing what he had done, and knowing that although he had sent for the doctor no doctor had appeared, should have "continued his duties until seven o'clock in the morning, when he was relieved"—all this seems to us a perfect nightmare of callousness and officialism. That an attendant should one night give an extra turn to the poker and kill his man is terrible enough, and no one probably will think that the punishment meted out to him is too great; but he was not wholly to blame. What about the system under which it was possible for him to "know how to quiet a troublesome man like that"—the system under which such a use of the poker was not enough to startle his assistant out of his routine and drive him to do something else than "go about his duties"? Clearly this is not the first time that patients have been submitted to torture by strangulation, and, unless we do something to reform the system under which such things can happen, it will not be the last.

